# Heart Rate Variability Analysis of Healthy Individuals and Patients with Ischemia and Arrhythmia

**DOI:** 10.3390/diagnostics13152549

**Published:** 2023-07-31

**Authors:** Galya Georgieva-Tsaneva, Evgeniya Gospodinova

**Affiliations:** Institute of Robotics, Bulgarian Academy of Science, 1113 Sofia, Bulgaria; jenigospodinova@abv.bg

**Keywords:** Holter monitoring, PPG signals, ECG signals, heart rate variability, cardiovascular diseases, mathematical analysis, arrhythmia, ischemic heart disease (IHD)

## Abstract

This article presents the results of a study of the cardiac activity of patients diagnosed with arrhythmia and ischemic heart disease. The obtained results were compared with the results obtained from a healthy control group. The studies were conducted on long-term cardiac recordings (approximately 24 h) registered by means of Holter monitoring, and the observations were made in the daily activities of the individuals. All processing, analysis and evaluations on the registered signals were performed by means of an established information demonstration cardiology system. The mathematical analysis included linear, non-linear and graphical methods for estimating and analyzing heart rate variability (HRV). Re-examinations were carried out on some of the observed individuals after six months of treatment. The results show an increase in the main time domain parameters of the HRV, such as the SDNN (from 86.36 ms to 95.47 ms), SDANN (from 74.05 ms to 82.14 ms), RMSSD (from 5.1 ms to 6.92 ms), SDNN index (from 52.4 to 58.91) and HRVTi (from 12.8 to 16.83) in patients with ischemia. In patients with arrhythmia, there were increases in the SDNN (from 88.4 ms to 96.44 ms), SDANN (from 79.12 ms to 83.23 ms), RMSSD (from 6.74 ms to 7.31 ms), SDNN index (from 53.22 to 59.46) and HRVTi (from 16.2 to 19.42). An increase in the non-linear parameter α (from 0.83 to 0.85) was found in arrhythmia; and in α (from 0.80 to 0.83), $\alpha_{1}$ (from 0.88 to 0.91) and $\alpha_{2}$ (from 0.86 to 0.89) in ischemia. The presented information system can serve as an auxiliary tool in the diagnosis and treatment of cardiovascular diseases.

## 1. Introduction

The human cardiovascular system is responsible for the overall functioning of the body, and its proper operation is of fundamental importance for the fulfillment of human life. The blood pumped by the heart circulates throughout the body and reaches every organ and every cell through a complete system of arteries, veins and capillaries, delivering oxygen and nutrients.

In today’s post-pandemic times, the realization of the task of the long-term monitoring of patients, which will guarantee disease control and the maintenance of people’s health, has come to the fore. Long-term monitoring makes it possible to detect cardiovascular problems that are rarely manifested in people’s daily activities but can pose a risk to the health and life of the individual. Holter monitoring is a modern method for obtaining quality health care for residents in small settlements where there are no hospital facilities and no possibility of a hospital stay at the place of residence. In completely remote and inaccessible places, these health services can be provided, thereby ensuring equal health services to all people.

Heart rate variability (HRV) is a non-invasive indicator that is obtained during the monitoring of the heart rhythm and provides information about the general state of health of the body. HRV parameters can provide a very useful report about the course of cardiovascular diseases and the patient’s health prognosis. For example, studies [1,2] show that after an MI, patients with an SDNN below 70 ms on a 24 h ECG recording have an almost four times higher chance of dying in the next 3 years.

HRV decreases in conditions of stress [3], with deterioration of cardiac activity, etc. At the same time, HRV assesses the parasympathetic balance in the body, which makes it possible to predict the development of cardiovascular diseases. The following values are accepted as normal in the elderly [4]: minimum heart rate of 60 bpm; maximum heart rate: of 100 bpm.

Of the cardiovascular diseases, arrhythmia is one of the most critical conditions [5] of the heart, for which a physiological rationale continues to be sought [6]. Several million people suffer from arterial fibrillation in the United States, and their number is constantly increasing [7,8], as is the mortality from this condition [9]. Among the consequences of acute infection with COVID-19 are diseases of the cardiovascular system, such as palpitations, cardiac arrhythmia and cardiopulmonary symptoms (chest pain, shortness of breath, tachycardia) [10,11]. Bradycardia has been documented in one third of affected patients following COVID-19 [12]. The high prevalence of cardiac arrhythmias requires their careful study [13] and the realization of means for their monitoring.

Ischemic heart disease is among the major cardiovascular diseases that cause high morbidity and mortality in the population [14] worldwide [15], mainly affecting the elderly population [16]. Eastern European countries (including Bulgaria) are among the most affected by this disease [17].

### The Purpose of This Article

Although in recent years there have been many publications related to the processing, analysis and evaluation of HRV, some problems still remain unsolved. For example, linear methods are standardized, but all other non-linear methods are not. The purpose of this article is to support the diagnosis of cardiovascular diseases such as arrhythmia and ischemic heart disease by applying mathematical methods in HRV analysis. To achieve this goal, the following two tasks are set:

1. The study of HRV using linear and non-linear methods on records including arrhythmia and ischemia in comparison with the records of healthy individuals.
2. The study of the changes occurring in HRV after treatment under medical supervision.

## 2. Linear Methods of Analysis

These methods are standardized [18]. Table 1 presents a description of the parameters and gives the normal values for the linear statistical parameters according to the accepted standard for HRV. Time domain methods represent a statistical calculation of consecutive RR intervals of the investigated cardio record. Their advantage is their easy determination and the availability of normalized values (these methods are standardized), which is why they have been widely used. Their sensitivity to the presence of artifacts in the recording can be cited as a limitation of their use.

## 3. Nonlinear Methods of Analysis

Detrended Fluctuation Analysis (DFA). This method [19] makes it possible to study the internal self-similar dynamics in non-stationary signals such as biomedical data and, in particular, cardiac data. DFA detects the short-term and long-term correlations in the studied data by the parameters $\alpha_{1}$ and $\alpha_{2}$. The studied signal is segmented and the method of least squares is applied, and the slope of the regression line is determined. The values of the alpha parameter are examined:

1. 0 < α< 0.5—presence of negative correlations;
2. α = 0.5—white noise;
3. 0.5 < α < 1—presence of self-similarity.

Poincaré method. With this popular geometric method [20], the following parameters are determined (Table 2):

- SD1 determines the short-term variability between heart beats (parasympathetic activity);
- SD2 describes long-term variations (sympathetic and parasympathetic activity);
- SD1/SD2 determines the randomness of the HRV signal.

**Table 2:** Nonlinear analysis parameters.

Parameter	Name	Description
DFA method		
α	Alpha parameter	Determine the total HRV correlation
$\alpha_{1}$	Alpha1 parameter	Determine the short-term HRV correlation
$\alpha_{2}$	Alpha2 parameter	Determine the long-term HRVcorrelation
Poincaré method		
SD1	Standard deviation (Ellipse length)	Determines short-term variability
SD2	Standard deviation (Ellipse width)	Describes long-term variations
SD1/SD2	Ellipse length/Ellipse width ratio	Determines the unpredictability of the time series
Entropies		
AppEn	Approximate Entropy	Determine the similarity and complexity of heart rate rhythms in a time series
SampEn	Sample Entropy	Determine variation in the time interval between consecutive heartbeats
R/S method		
h	Hurst exponent	Determines the self-similarity in the time series

Entropy. In a number of research works [21,22,23], the non-linear parameters AppEn and SampEn (Table 2) are used—a commonly used tool for analyzing the regularity/irregularity of time series.

Self-similarity. The presence of fractality and self-similarity were investigated by determining the Hurst exponent h (Table 2).

## 4. Graphical Methods for HRV Analysis

These methods include spectrograms, which give a time–frequency dependence of the distribution of spectral frequencies. In the spectrogram method, the power spectral density (PSD) (located along the vertical axis in the graph) is examined as a function of the frequency (along the horizontal axis in the graph).

Through the methods of frequency analysis, information is obtained about the way the spectrum is distributed in the frequency domain, without being able to determine when exactly in time the frequencies appear, how long a period of time they refer to, etc. The method of time–frequency analysis allows the localization of the spectral components simultaneously in the frequency and time domain. The main technologies used for the time–frequency analysis were the fast Fourier transform window technique (short-time Fourier transform) and discontinuous wavelet transform.

When implementing the spectrogram method, window technology is applied. It divides the data time series into overlapping or non-overlapping consecutive segments for which the signal power spectral density is determined.

In recent years, wavelet analysis is often applied to analyze the local variations of the frequency spectrum in time series. The wavelet transform is suitable for studying the non-stationary HRV signal, as it not only provides insight into the distribution of spectral frequencies but also shows exactly where these frequencies appear and disappear along the time axis. By representing the time series in the time–frequency domain, the variability of the data under study can be more accurately examined as to how the variability changes over time. The wavelet method uses interpolation (with a specified frequency) with a wavelet transform and a selected basis and calculates a continuous wavelet spectrum.

## 5. The Demographic Characteristics

The data used for the research in this article were recorded with a Dynamic ECG Systems TLC9803 Holter device at the Medical University of Varna, Bulgaria. A cardiologist was involved in the registration of the data, who made the relevant diagnoses and treated the subjects. The demographic characteristics of the studied records of the healthy individuals and arrhythmia patients diagnosed are presented in Table 3. Research was conducted on previously anonymized records. Table 3 provides information on age (average age of individuals) and provides a gender distribution. The studied RR time series are of 122 subjects, which are united in the following three groups:

- Group 1—patients with ischemia, which consists of 16 patients, including 6 men and 10 women. The average age of the group is 37.50 years.
- Group 2—patients with arrhythmia, which consists of 64 patients, including 28 men and 36 women. The average age of the group is 43.75 years.
- Group 3—healthy subjects, which consists of 42 subjects, including 20 men and 22 women. The average age for this group is 47.62 years.

**Table 3:** Table of demographic characteristics.

Parameters	Patients with Ischemia *N* = 16	Patients with Arrhythmia *N* = 64	Healthy Subjects *N* = 42	*p*-Value (Ischemia/ Healthy	*p*-Value (Arrhythmia /Healthy
Gender, Men [%]	37.50	43.75	47.62	NS ^1^	NS ^1^
Age ± sd	42.25 ± 15	49.14 ± 14.16	44.65 ± 10.52	NS ^1^	NS ^1^

^1^ NS—not significant.

There is no significant difference between the healthy, ischemic heart disease and arrhythmia groups according to their demographic characteristics.

The group corresponding to patients with arrhythmia includes only subjects with supraventricular extrasystoles. The treatment of patients from this group was carried out by taking antiarrhythmic drugs, including beta-selective blockers.

The group corresponding to patients with ischemic disease includes only patients whose treatment includes vasodilator drugs, including beta blockers. Research has shown that patients who have undergone percutaneous coronary angiography with an implanted stent have, as a result of the treatment, changes in heart rate and HRV values approaching those of the control group. These results are seen almost immediately after placement of the stent.

Some of the study participants were re-examined after six months of treatment to monitor the change in HRV parameters.

All participants in the conducted research signed an informed consent form. They received explanations about the nature and purpose of the study, as well as about the procedures and inconveniences associated with it. Participants were given the opportunity to ask questions and by signing they agreed to voluntarily participate in this study.

The 24 h recordings, consisting of about 100,000 RR intervals, recorded with the Holter device were analyzed using Matlab experimental software.

## 6. Results

The created demonstration software system can work with all three types of recordings—ECG, PPG and Holter. The studies on their derived HRV time series are exactly the same. In the rest of the article, the presented studies were made with the information system on long-term (24 h) records registered with Holter monitoring.

**Figure 1:**
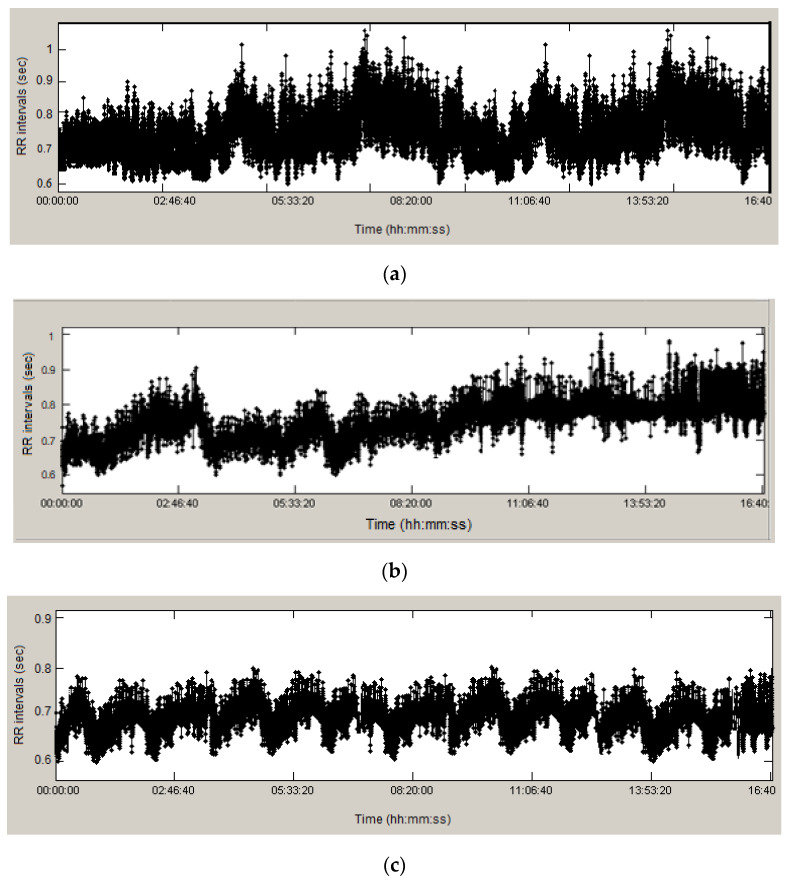
RR intervals of: (**a**) healthy (**b**) arrhythmic; and (**c**) ischemic patients.

Figure 1 shows the obtained time series of RR intervals from recordings of a healthy individual (Figure 1a); the values of RR intervals vary widely from 0.6 s to 1.1 s, and the differences between adjacent intervals also vary considerably. The presented graph of a patient diagnosed with arrhythmia (Figure 1b) varies from 0.6 s to 1.0 s, and the graph in Figure 1c (patient diagnosed with ischemic heart disease) varies from 0.6 s to 0.81 s. The graphs were obtained using the developed software system for processing and analysis. Of interest are the repeated segments in the ischemia recording, indicating the presence of self-similarity.

**Figure 2:**
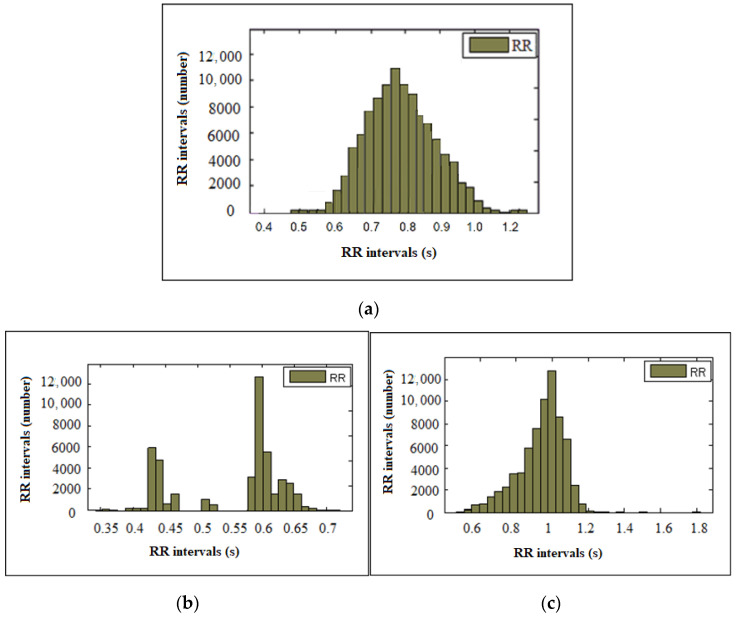
Histogram of RR intervals of: (**a**) a healthy individual; (**b**) a patient with arrhythmia; and (**c**) a patient with ischemia.

Figure 2 shows the histograms of a healthy individual (Figure 2a), an arrhythmic patient (Figure 2b) and an ischemic patient (Figure 2c). The histogram of a healthy individual has the characteristic shape of a normal Gaussian distribution—a symmetric bell shape—and the intervals have a central mode of 0.75 s. In the patient with arrhythmia, there is a concentration of intervals around two modes—0.6 s and 0.42 s. In the patient with ischemia, the histogram is shifted to the left, presenting asymmetry.

**Figure 3:**
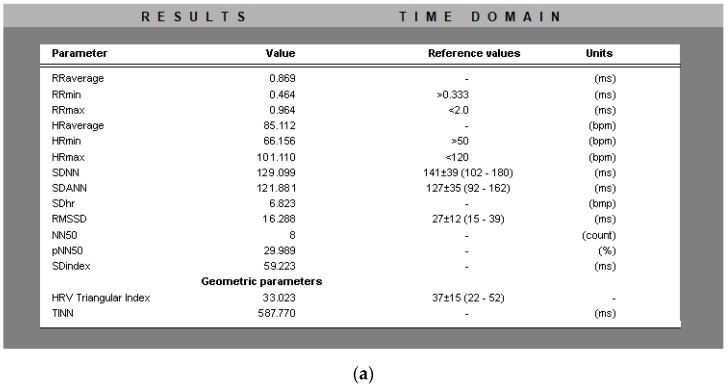

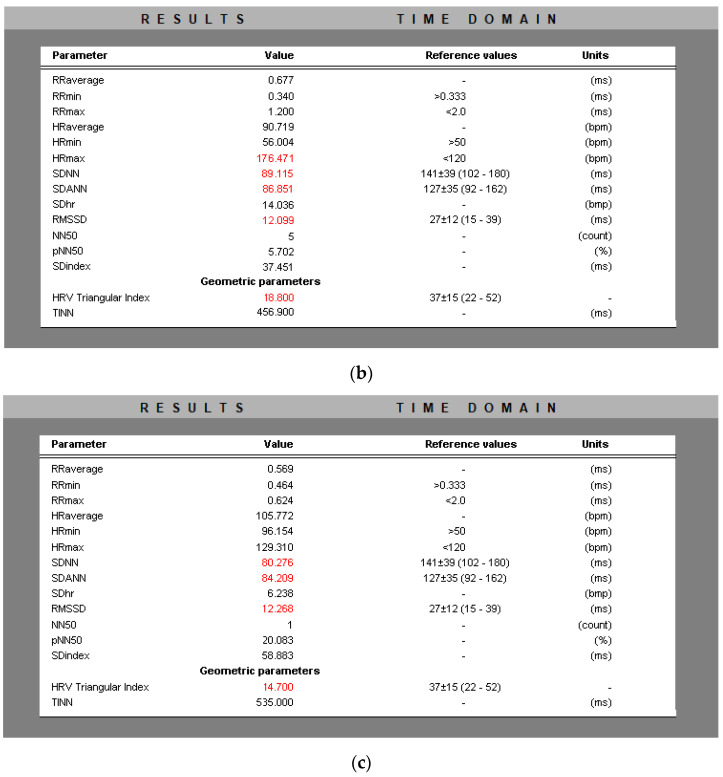
Results in the time area of: (**a**) a healthy individual; (**b**) a patient with arrhythmia; and (**c**) a patient with ischemia. The values of the parameters that are not within the limits of normal for a healthy individual are colored in red.

Figure 3 presents results in the time domain of a patient with ischemia. The table in the figure is the result of running the created HRV analysis program. The values of the parameters that are outside the norms are displayed in red.

Table 4 presents the results of the time domain analysis performed on the two types of recordings of patients with arrhythmia and healthy subjects. Values are expressed as the mean ± standard deviation (sd) or as percentages.

The research shows that the mean value of MeanRR in healthy subjects is 889.22 ms, and it is higher than the mean value of MeanRR (732.31 ms) obtained for patients with arrhythmia, as well as for patients diagnosed with ischemic heart disease (710.44 ms). The SDNN parameter for the arrhythmia group (mean value: 91.23 ms) is lower compared to the SDNN calculated in the healthy group (mean value: 132.55 ms) but not compared to the values of the parameter in the group with ischemia; in this group, the parameter is even lower (88.36 ms). The same is observed for the SDANN parameter (81.56 ms in the arrhythmia group and 77.05 ms in the ischemia group versus 128.51 ms in the healthy control group). The RMSSD parameter is significantly lower in the diseased subjects compared to the healthy control group (6.92 ms in arrhythmia and 5.88 ms in IHD versus 16.42 ms in healthy subjects). The same is observed with the parameters of the SDNN index and HRVTi. The pNN50 score is higher in the diseased subjects compared to the healthy control group (a mean value of 39.18 in arrhythmia versus a mean value of 28.72 in the control group). This indicates that in healthy subjects with normal sinus rhythm, the total number of intervals less than 50 ms in length is smaller, which is due to the fact that the duration of maximal RR intervals is usually well over 50 ms.

Table 5 presents the investigated parameters for DFA, the Poincaré method, entropies and the Hurst exponent. Nonlinear DFA parameters (α, $\alpha_{1}$, $\alpha_{2}$) were lower in arrhythmia recordings compared to the corresponding parameters in healthy subjects’ recordings. The table presents the average values of a total of 16 recordings with IHD, 64 recordings with arrhythmia and 42 recordings of healthy subjects. The Hurst exponent was calculated using the R/S statistical method. In recordings with arrhythmia, h has a mean value of 0.96, which is significantly higher than the value of 0.77 in healthy subjects.

**Figure 4:**
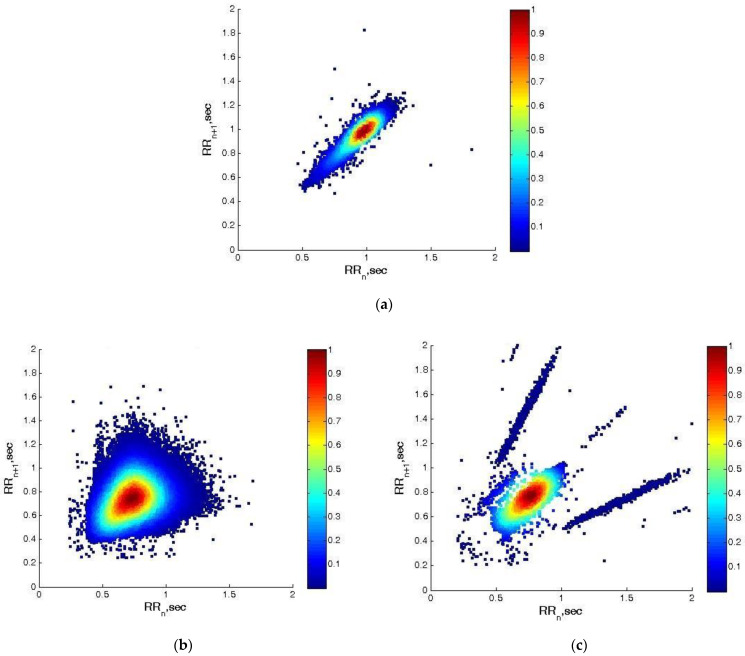
Poincaré method of the RR intervals of: (**a**) a healthy individual; (**b**) a patient with arrhythmia; and (**c**) a patient with ischemia.

Figure 4 presents the graphs of the studied groups using the Poincaré graphical method. The graph of the healthy individual has the characteristic comet shape, tapered at the bottom and slightly widening at the top. In the patient with arrhythmia, the graph is very widened at its upper right edge and in this particular case has a triangle shape. The plot of the IHD patient is dotted and shows a very large irregularity in the studied time series.

**Figure 5:**
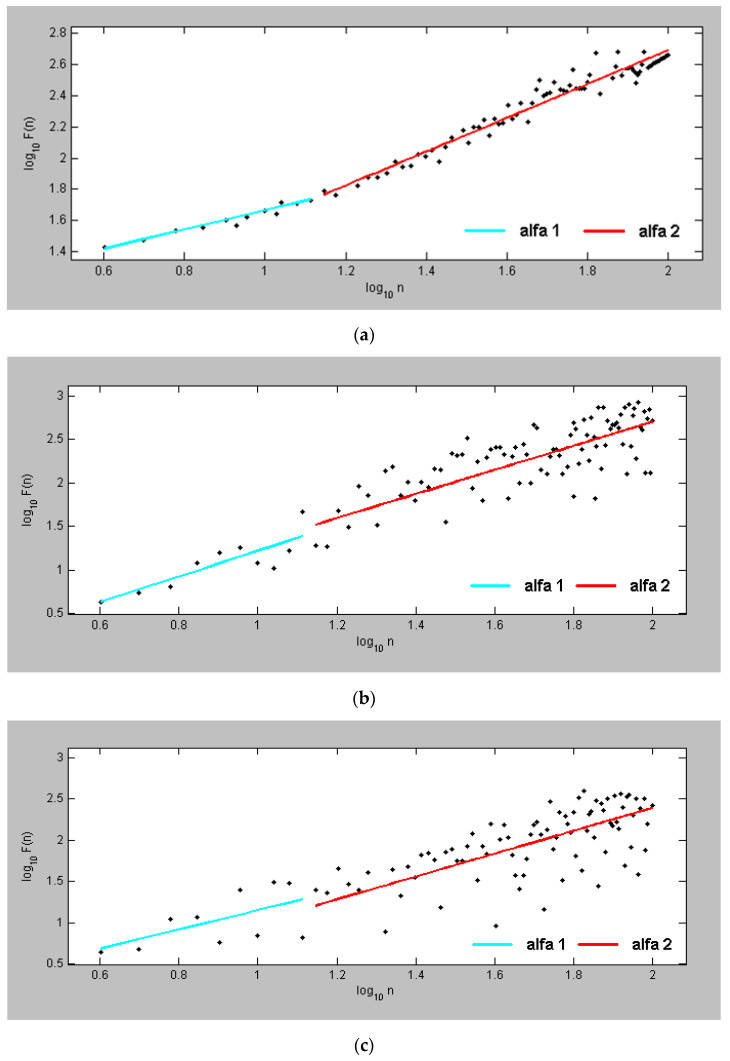
DFA for (**a**) a healthy individual; (**b**) a patient with arrhythmia; and (**c**) a patient with IHD.

Figure 5a presents the two parameters $\alpha_{1}$ and $\alpha_{2}$ of the DFA for a Holter recording of a healthy person. The obtained value is $\alpha_{1}$ = 1.14 for the short correlations and $\alpha_{2}$ = 1.32 for long correlations. The conducted studies show higher values for the parameter $\alpha_{2}\;\mathrm{relative}\;\mathrm{to}\;\alpha_{1}.$

Figure 5b presents the two parameters $\alpha_{1}$ and $\alpha_{2}$ for the arrhythmia Holter recording. The obtained parameter is $\alpha_{1}$ = 0.94 for short correlation and $\alpha_{2}$ = 0.92 for long correlation. Figure 5b shows that the two investigated parameters have similar values in the arrhythmia recording.

The comparison of the values of the two studied parameters in the healthy individual and in the patient with arrhythmia shows lower values of these parameters in the recording with arrhythmia. Figure 5c shows the DFA plot in a patient with ischemia. Low values of the studied parameters are observed, scattered around the trend line, but with a significantly lower variation than in the records of healthy subjects.

**Figure 6:**
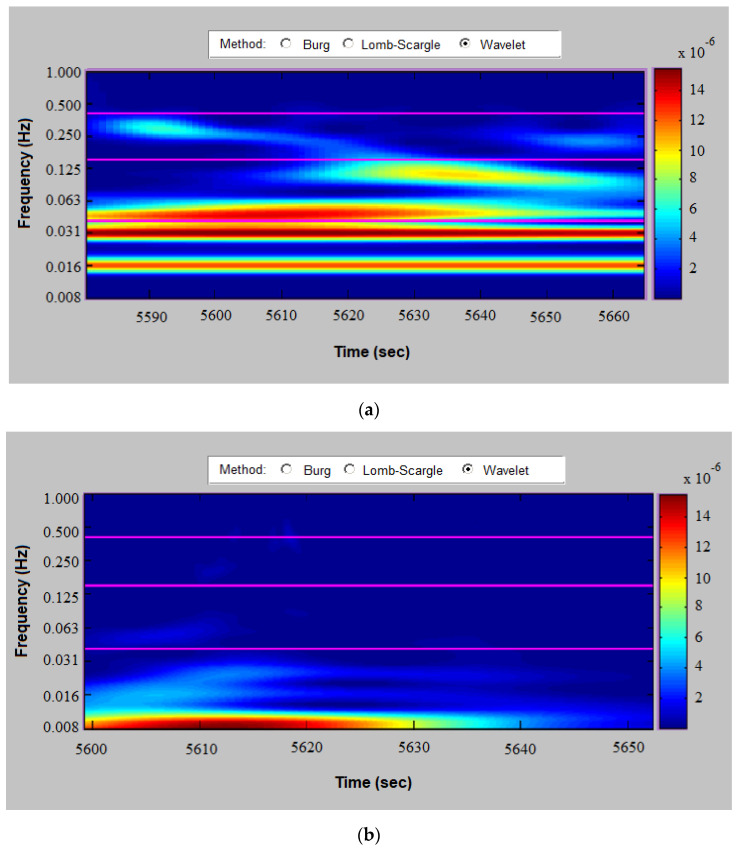

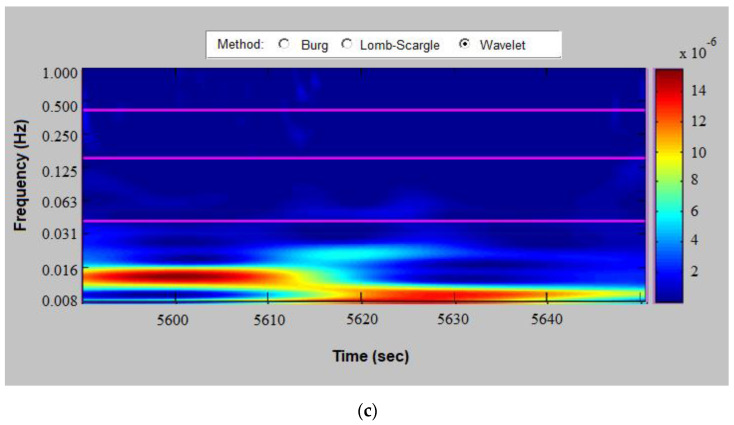
Spectrogram for (**a**) a healthy individual; (**b**) a patient with arrhythmia; and (**c**) a patient with IHD.

The following figures show spectrograms (using a continuous wavelet transform implementation) of a healthy individual (Figure 6a), an arrhythmic patient (Figure 6b) and an ischemic patient (Figure 6c). The depicted spectrogram of individuals with cardiovascular disease shows low signal power values throughout the high-frequency range and predominantly low signal power values in the low-frequency range. Low frequency values in these two ranges indicate a low heart rate variability, which is an indicator of poor health.

The monitoring of indicators with adequate treatment under the supervision of a cardiologist was conducted on a proportion of the studied patients. The trends in the treatment of the diseases were studied.

After six months of treatment, the investigated HRV indicators show an increasing trend. The most sensitive increase is observed in time domain indicators (SDNN, SDANN, RMSSD, pNN50, and SDNN index). Non-linear indicators increase less, which indicates that these indicators probably change more slowly when the body’s health status changes.

Table 6 presents the results of the analyses performed on four patients with ischemia and three patients with arrhythmia before and after 6 months of treatment. An increase in the values of the HRV parameters in the time domain and of the non-linear indicators is observed in the studies conducted after the six-month treatment conducted under medical supervision. A decrease in the value of the Hurst exponent was reported after treatment.

### Statistical Analysis

The T test method was used for statistical analysis. In this study, if the *p*-value is less than or equal to 0.05 (5%), the result is considered statistically significant.

Statistical analysis of the geometric parameters shows that the geometric parameter HRVTi (Table 4) is lower in the diseased subjects compared to the healthy control group (16.53 in arrhythmia versus 31.17 in the healthy individuals) and reaches statistical significance (*p*-value < 0.01). TINN (464.1 ms in the arrhythmia group vs. 508.42 ms in the control healthy group) has no statistical significance (*p* > 0.05).

Table 4 shows that for the determined values for the studied parameters (without TINN), the value of the parameter *p* < 0.05 is selected as an indicator of their statistical significance.

## 7. Discussion

Linear methods have been well studied and are considered suitable when testing heart rate variability. The methods reviewed and researched in this article are the basis for creating a software information platform using modern tools to support the diagnosis, prognosis and prevention of cardiovascular diseases.

The research conducted and the results presented show reduced values of heart rate variability indicators in patients with arrhythmia and IHD compared to healthy people. In the time domain, the parameters SDNN, SDANN, RMSSD, pNN50 and the HRV triangular index were lower in the examined cardiac data of diseased subjects compared to healthy subjects and were significantly lower than their respective reference values (presented in [5]).

An additional means of studying the recorded cardio signals are non-linear methods, which are in the process of study and have not been standardized thus far. The parameters $\alpha ,\alpha_{1},\alpha_{2}$ (DFA) and h (R/S method) are lower in the studied patients with cardiovascular diseases compared to healthy people.

In addition to the numerical results, the obtained graphical results are also of interest, which can give an overview of the health status of the individuals. The histogram in healthy individuals has the form of a normal Gaussian distribution; while in the studied individuals with arrhythmia and IHD, the histograms are completely different from this type of distribution, and in different diseases they can have a different graphic appearance.

The application of non-linear graphical analyses of HRV is an effective method for visualizing the fluctuations of the pulse frequency interval series. From the obtained results, detailed information about the condition of patients can be extracted, which allows doctors to monitor the course of the disease. The graphs obtained by the Poincaré method graphically illustrate the nature of the studied cardio time series. In a healthy individual, the graph has the shape of a comet, represented in the examined patients as a very wide upper right edge; in patients with IHD, the points are chaotically scattered and show an irregularity of the cardio series.

Arrhythmia is a condition in which the heart beats abnormally (out of normal rhythm). In many cases it is harmless but in some it can be life-threatening. Ischemic heart disease is a narrowing or occlusion of one or more coronary arteries with subsequent myocardial ischemia, which occurs with or without attacks of pain behind the sternum. It is one of the most common heart diseases, characterized by damage to the heart as a result of insufficient oxygen supply to the organ.

The conducted studies show the strongest reduction in HRV indicators in the time domain (SDNN, SDANN, RMSSD, pNN50, SDNN index), as well as in the non-linear analyses performed ($\alpha ,\alpha_{1},\alpha_{2},SD1,SD2,AppEn,SampEn)$ for patients with ischemic heart disease. The obtained results show the significance of this disease for individuals and the need for timely and adequate treatment.

### 7.1. Limitations

The research conducted has certain limitations related to the small number of Holter recordings that were examined (12 records of individuals diagnosed with IHD, 64 records with arrhythmia and 42 records of healthy individuals). The database (http://hrvdata.vtlab.eu (accessed on 25 July 2023)) is about to be completed.

### 7.2. Future Directions

As a future activity, the authors envisage the use of 3D technologies [24] and virtual reality to induce stressful situations in healthy individuals and study HRV in such situations, which is common in the daily life of modern people.

## 8. Conclusions

Research shows that after six months of treatment conducted by a cardiologist, the time domain parameters measured for the observed patients changed significantly (*p*-value < 0.05). The changes were also observed in the group of patients diagnosed with ischemic heart disease and in the group with arrhythmia. No significant changes were observed in the parameters of the non-linear methods, but a tendency to improve the values of the HRV parameters was observed.

The assessment of the set of HRV indicators makes it possible to improve the treatment carried out and periodically monitor the health indicators of the patients, and this approach can lead to the improvement of the treatment process.

## Figures and Tables

**Table 1:** Reference values of parameters in the time domain [18].

Parameter	Description	Formula	Healthy Subjects	Unit
RRmin	Minimum interval length	-	>0.333	ms
RRmax	Maximum length of intervals	-	<2.0	ms
SDNN	Standard deviation (sd) of RR intervals	$\sqrt{\frac{1}{N}\sum_{i=1}^{N}\left({RR}_{i}-\bar{RR}\right)}$	102–180	ms
SDANN	Standard deviation of consecutive 5 min intervals	$\sqrt{\frac{1}{N}\sum_{i=1}^{N}({{\bar{{RR}_{i}}}_{}-\overset{=}{RR})}^{2}}$	92–162	ms
RMSSD	Square root of the mean squared differences between consecutive cardio intervals	$\sqrt{\frac{1}{N-1}\sum_{i=1}^{N-1}({{RR}_{i+1}-\bar{{RR}_{i}})}^{2}}$	15–39	ms
pNN50	NN50 divided by the total number of cardio intervals	$pNN50=\frac{NN50}{N}\cdot 100$	-	%
SDNN Index	Mean of the sd of all cardio intervals for each five-minute block (determined over a 24 h recording)	$\frac{1}{N}\sum_{i=N}^{N}S{DNN}_{i}$	-	ms
HRVTi	Given by the most frequent value X (mode) with its absolute frequency Y (based on a histogram of cardio intervals)	$\frac{\sum_{i=1}^{N_{b}}b\left(t_{i}\right)}{{max}_{i}b\left(t_{i}\right)}=\frac{N-1}{{max}_{i}b(t_{i})}$	22–52	-
TINN	Truangular Interpolation of Normal_to_Normal interval histogram. Describes total variations of HR	Calculated from the difference triangular interpolation of the maximum of the intervals sample density distribution	-	ms

**Table 4:** Time domain parameters (results).

Parameters	Patients with Ischemia *N* = 16 (Mean ± sd)	Patients with Arrhythmia *N* = 64 (Mean ± sd)	Healthy Subjects *N* = 42 (Mean ± sd)	*p*-Value (Ischemia/ Healthy)	*p*-Value (Arrhythmia /Healthy)
MeanRR (ms)	710.44 ± 161.53	732.31 ± 142.65	889.22 ± 128.06	<0.01	<0.05
SDNN (ms)	88.36 ± 31.06	91.23 ± 23.97	132.55 ± 64.91	<0.001	<0.001
SDANN (ms)	77.05 ± 18.06	81.56 ± 52.24	128.51 ± 21.47	<0.005	<0.005
RMSSD (ms)	5.88 ± 10.83	6.92 ± 11.02	16.42 ± 6.42	<0.001	<0.001
pNN50	41.06 ± 3.19	39.18 ± 4.55	28.72 ± 6.95	<0.001	<0.001
SDNN Index (ms)	51.22 ± 21.11	52.13 ± 26.75	68.07 ± 11.93	<0.01	<0.01
HRVTi	14.48 ± 8.26	16.53 ± 12.98	31.17 ± 3.1	<0.005	<0.01
TINN [ms]	453.84 ± 115.29	464.1 ± 196.77	508.42 ± 212.61	NS ^1^	NS ^1^

^1^ NS—not significant.

**Table 5:** Nonlinear analysis parameters (results).

Parameters	Patients with Ischemia *N* = 16 (Mean ± sd)	Patients with Arrhythmia *N* = 64 (Mean ± sd)	Healthy *N* = 42 (Mean ± sd)	*p*-Value (Ischemia/ Healthy	*p*-value (Arrhythmia /Healthy
DFA method					
α	0.79 ± 0.41	0.83 ± 0.34	1.16 ± 0.55	<0.01	<0.05
$\alpha_{1}$	0.89 ± 0.32	0.91 ± 0.63	1.11 ± 0.37	<0.001	<0.005
$\alpha_{2}$	0.86 ± 0.37	0.94 ± 0.41	1.28 ± 0.24	<0.001	<0.001
Poincaré method					
SD1 (ms)	0.64 ± 0.23	0.71 ± 0.08	0.85 ± 0.41	<0.05	<0.05
SD2 (ms)	0.81 ± 0.31	0.89 ± 0.32	0.98 ± 0.34	<0.05	<0.05
SD1/SD2	0.33 ± 0.02	0.28 ± 0.02	0.14 ± 0.01	<0.05	<0.05
Entropies					
AppEn	1.02 ± 0.14	1.22 ± 0.18	1.58 ± 0.05	<0.001	<0.001
SampEn	1.12 ± 0.16	1.26 ± 0.23	1.56 ± 0.14	<0.001	<0.001
R/S method					
h	0.97 ± 0.48	0.96 ± 0.31	0.77 ± 0.24	<0.01	<0.01

**Table 6:** HRV parameters before and after treatment.

Parameters	Patients with Ischemia (before Treatment) *N* = 4 (Mean ± sd)	Patients with Arrhythmia (before Treatment) *N* = 3 (Mean ± sd)	Patients with Ischemia (after Treatment) *N* = 4 (mean ± sd)	Patients with Arrhythmia (after Treatment) *N* = 3 (Mean ± sd)	*p*-Value (Changes before vs. after Treatment, Ischemia)	*p*-Value (Changes before vs. after Treatment, Arrhythmia)
MeanRR (ms)	705.55 ± 160.1	732.35 ± 180.65	710.09 ± 184.05	738.02 ± 122.23	NS ^1^	NS ^1^
SDNN (ms)	86.36 ± 4.06	88.4 ± 5.22	95.47 ± 5.15	96.44 ± 4.32	<0.05	<0.05
SDANN (ms)	74.05 ± 18.06	79.12 ± 22.05	82.14 ± 12.31	83.23 ± 22.06	<0.05	NS
RMSSD (ms)	5.1 ± 1.02	6.74 ± 1.35	6.92 ± 1.24	7.31 ± 1.04	<0.05	<0.05
pNN50	10.05 ± 3.05	13.33 ± 2.02	15.61 ± 3.42	17.44 ± 2.09	<0.05	<0.05
SDNN Index (ms)	52.40 ± 3.3	53.22 ± 3.18	58.91 ± 4.14	59.46 ± 4.04	<0.05	<0.05
HRVTi	12.8 ± 2.3	16.2 ± 3.22	16.83 ± 2.22	19.42 ± 2.05	<0.05	NS ^1^
TINN [ms]	437.15 ± 22.85	445.02 ± 21.2	482.09 ± 29.11	492.71 ± 31.02	<0.05	<0.05
DFA method						
α	0.80 ± 0.25	0.83 ± 0.34	0.83 ± 0.32	0.85 ± 0.22	NS ^1^	NS ^1^
$\alpha_{1}$	0.88 ± 0.12	0.91 ± 0.63	0.91 ± 0.11	0.92 ± 0.71	NS ^1^	NS ^1^
$\alpha_{2}$	0.86 ± 0.05	0.94 ± 0.41	0.89 ± 0.34	0.95 ± 0.82	NS ^1^	NS ^1^
R/S method						
h	0.97 ± 0.25	0.96 ± 0.13	0.94 ± 0.1	0.92 ± 0.5	NS ^1^	NS ^1^

^1^ NS—not significant.

## Data Availability

The cardio data we processed for the research purposes of this paper were obtained from the Medical University of Varna, Bulgaria (available on http://hrvdata.vtlab.eu/, (accessed on 25 January 2023)).
